# Supplementary material for: Time-series transcriptome analysis identified differentially expressed genes in broiler chicken infected with mixed Eimeria species
Source: Front Genet. 2022 Aug 8;13:886781. doi: 10.3389/fgene.2022.886781 (PMC9393255; doi:10.3389/fgene.2022.886781)
Supplement: Supplementary file 2 [file DataSheet1.ZIP › 7dpi_KEGG.Gsea.1625115961950/KEGG_PENTOSE_AND_GLUCURONATE_INTERCONVERSIONS.html]

Details for gene set KEGG\_PENTOSE\_AND\_GLUCURONATE\_INTERCONVERSIONS[GSEA]

|  || Dataset | TMM\_7dpi\_gct\_format\_7dpi\_gct\_format.Class\_4dpi.cls #PC\_versus\_NC.Class\_4dpi.cls #PC\_versus\_NC\_repos |
| Phenotype | Class\_4dpi.cls#PC\_versus\_NC\_repos |
| Upregulated in class | 1 |
| GeneSet | KEGG\_PENTOSE\_AND\_GLUCURONATE\_INTERCONVERSIONS |
| Enrichment Score (ES) | 0.49003628 |
| Normalized Enrichment Score (NES) | 1.4423949 |
| Nominal p-value | 0.07765452 |
| FDR q-value | 0.20148765 |
| FWER p-Value | 0.7888 |
Table: GSEA Results Summary

  

Fig 1: Enrichment plot: KEGG\_PENTOSE\_AND\_GLUCURONATE\_INTERCONVERSIONS      
 Profile of the Running ES Score & Positions of GeneSet Members on the Rank Ordered List

  

| SYMBOL | TITLE | RANK IN GENE LIST | RANK METRIC SCORE | RUNNING ES | CORE ENRICHMENT || 1 | UGDH | na | 117 | 1.206 | 0.2005 | Yes |
| 2 | AKR1B1 | na | 535 | 0.720 | 0.2913 | Yes |
| 3 | RPEL1 | na | 663 | 0.653 | 0.3945 | Yes |
| 4 | UGT1A1 | na | 772 | 0.599 | 0.4900 | Yes |
| 5 | DCXR | na | 2596 | 0.179 | 0.3693 | No |
| 6 | DHDH | na | 4357 | -0.036 | 0.2289 | No |
| 7 | GUSB | na | 6690 | -0.274 | 0.0824 | No |
| 8 | UGP2 | na | 7288 | -0.339 | 0.0918 | No |
| 9 | UGT2A1 | na | 7912 | -0.410 | 0.1113 | No |
| 10 | XYLB | na | 9680 | -0.657 | 0.0787 | No |
| 11 | CRYL1 | na | 9706 | -0.661 | 0.1920 | No |
Table: GSEA details [plain text format]

  

Fig 2: KEGG\_PENTOSE\_AND\_GLUCURONATE\_INTERCONVERSIONS      
 Blue-Pink O' Gram in the Space of the Analyzed GeneSet

  

Fig 3: KEGG\_PENTOSE\_AND\_GLUCURONATE\_INTERCONVERSIONS: Random ES distribution      
 Gene set null distribution of ES for **KEGG\_PENTOSE\_AND\_GLUCURONATE\_INTERCONVERSIONS**
